# 

**Published:** 1857-06

**Authors:** 


### CONTENTS, No. 1, Vol. IV.

ORIGINAL COMMUNICATIONS. Page

Gun Shot Wound, Death, Autopsy, Geo. Shedd, M. D. - 1
Medical Properties of the May Apple, W. H. Letherman, M. D. 9
Poisoning by Strychnine, J. Ward Davidson, M. D. - - 11
Veratrum Viride, Prof, D. L. M'Gugin, M. D. - - 13
Fatty Placenta, Reply to Letter. - - - - - 20
Surgical Cases, Prof. J. C. Hughes. - - - - 21

DEPARTMENT OF SELECTIONS.

MEDICAL SOCIETIES. Page

Louisa Co. Med. Sc'y. - 51
Scott Co. Med. Sc'y. - 52
Am. Med. Ass. letter from Senior Editor. - - 53
Proceedings of Tenth Annual Session at Nashville. 56

EDITORIAL DEPARTMENT.

Surreptitious M. D's. - 70
Valedictory, Prof. M'Gugin. 73
Change of Editors. 74
Pecuniary Affairs of Journal. 75
State Medical Society. - 76
Our State Medical College. 76
New Books. - - - 79
Trans. Am. Med. Ass. 1856 79
Army Medical Appointments. 79
Receipts for Journal. - 79
Necrology. - - - 79
Miscellaneous Items. - 80

